# Supplementary material for: Development of a Monoclonal Antibody to a Vibriophage as a Proxy for Vibrio cholerae Detection
Source: Infect Immun. 2022 Jul 18;90(8):e00161-22. doi: 10.1128/iai.00161-22 (PMC9387236; doi:10.1128/iai.00161-22)
Supplement: Supplemental file 1 — Fig. S1 and S2; Tables S1 to S3. Download iai.00161-22-s0001.pdf, PDF file, 0.4 MB [file iai.00161-22-s0001.pdf]

SUPPLEMENTARY MATERIALS

Figure S1

A.

```
ORF75_ICP1_Bd_Protein       MNFSFLDMSLTEGYTEEYKKRYLEWKDGIPARITSTRDYESEQCVAVEFMIKDIYTWKGG     60
ORF75_ICP1_Goma_Protein     MNFSFLDMSLTEGYTEEYKKRYLEWKDGIPARITSTRDYETEQCVAVEFMIKDIYTWKGG     60
                            ****************************************:*******************

ORF75_ICP1_Bd_Protein       EDLRAVKLNKVFVRLPKFGPWVVKLPCSVDDLVILHFSSKDLNQFLAGNGEQVTQKAAEI     120
ORF75_ICP1_Goma_Protein     EDLRAVKLNKVFVRLPKFGPWVVKLPCSVDDLVILHFSSKDLNQFLAGNGEQVTQKAAEI     120
                            ************************************************************

ORF75_ICP1_Bd_Protein       GELEDCYAELGFGTRKSNNQPSLENLIVTNGAFTMTVTPQGDYTITTSGTGTYQAQKHTF     180
ORF75_ICP1_Goma_Protein     GELEDCYAELGFGTRKSNNQPSLENLIITNGAFTMTVTPQGDYTIITSGTGTYQAQKHTF     180
                            ***************************:***************** **************

ORF75_ICP1_Bd_Protein       KNDVEVEGNLTVKQNATVDGTITSKAGMFSPTYSGYGGAGSMTIGTITAQTSVTINGIEV     240
ORF75_ICP1_Goma_Protein     KNDVEIEGNLTVKQNTTVDGTVTSKAGMFSPTYSGYGGAGSMTIGTITAQTSVTIDGIEV     240
                            *****:*********:*****:*********************************:****

ORF75_ICP1_Bd_Protein       LGHKHTNPEGGDVGPMK   257
ORF75_ICP1_Goma_Protein     LGHNHTNPEGGDVGPMK   257
                            ***:*************
```

B.

```
ORF122_ICP1_Bd_Protein      MARMGDFGVVDYTSLMALAPRSKNFLELLGVFSESNTRYIDSRYAEFEREEKGVTKMNAM     60
ORF122_ICP1_Goma_Protein    MARMGDFGVVDYTSLMALAPRSKNFLELLGVFSESNTRYIDSRYAEFEREEKGVTKMNAM     60
                            ************************************************************

ORF122_ICP1_Bd_Protein      ARGGSRKYIGSEKARKEIIEVPFAPLDGVTVASEVEAFRQYGTESQTASIEALVQRKIEH     120
ORF122_ICP1_Goma_Protein    ARGGSRKYIGSEKARKEIIEVPFAPLDGVTVASEVEAFRQYGTESQTASVEALVQRKIEH     120
                            *************************************************:**********

ORF122_ICP1_Bd_Protein      IQRSHGIYIRDCQYTALLEDKILAEDEDGNEITALAKNFSTLWGVSRKTGAINTTTAVNP     180
ORF122_ICP1_Goma_Protein    IQRSHGIYIRDCQYTALLEDKILAEDEDGNEITALAKNFSTLWGVSRKTGAINTTTAVNP     180
                            ************************************************************

ORF122_ICP1_Bd_Protein      FSVLATKRQEIIDSMGENNGFTSMVVLCTTRDFNAIVDHPDVRAAYEGRDGGAEYLTRRL     240
ORF122_ICP1_Goma_Protein    FSVLATKRQEIIDSMGENNGFTSMVVLCTTRDFNAIVDHPDVRAAYEGRDGGAEYLTRRL     240
                            ************************************************************

ORF122_ICP1_Bd_Protein      GDAVDFQVFTHKGVTLVEDTSGKLTDGSAYMFPLGVQDMFQAVYAPADSTDHVNTISQGS     300
ORF122_ICP1_Goma_Protein    GDAVDFQVFTHKGVTLVEDTSGKLTDGSAYMFPLGVQDMFQAVYAPADSTDHVNTISQGS     300
                            ************************************************************

ORF122_ICP1_Bd_Protein      YLFLNAGENWRRDVIESEVSYACMVTRSELICDLTITVA   339
ORF122_ICP1_Goma_Protein    YLFLNAGENWRRDVIESEVSYACMVTRSELICDLTITVA   339
                            ***************************************
```

Fig S1. Alignment of Bangladesh (Bd) (14) and Goma ICP1 (19) bacteriophage ORF75 (**A**) and ORF122 (**B**) protein sequences by Clustal Omega (EMBL-EBI). Alignment is shown by symbols. The asterisk symbol represents identical amino acid, colon represents similar amino acid and any gaps in the alignment represent mismatched amino acid.

Figure S2

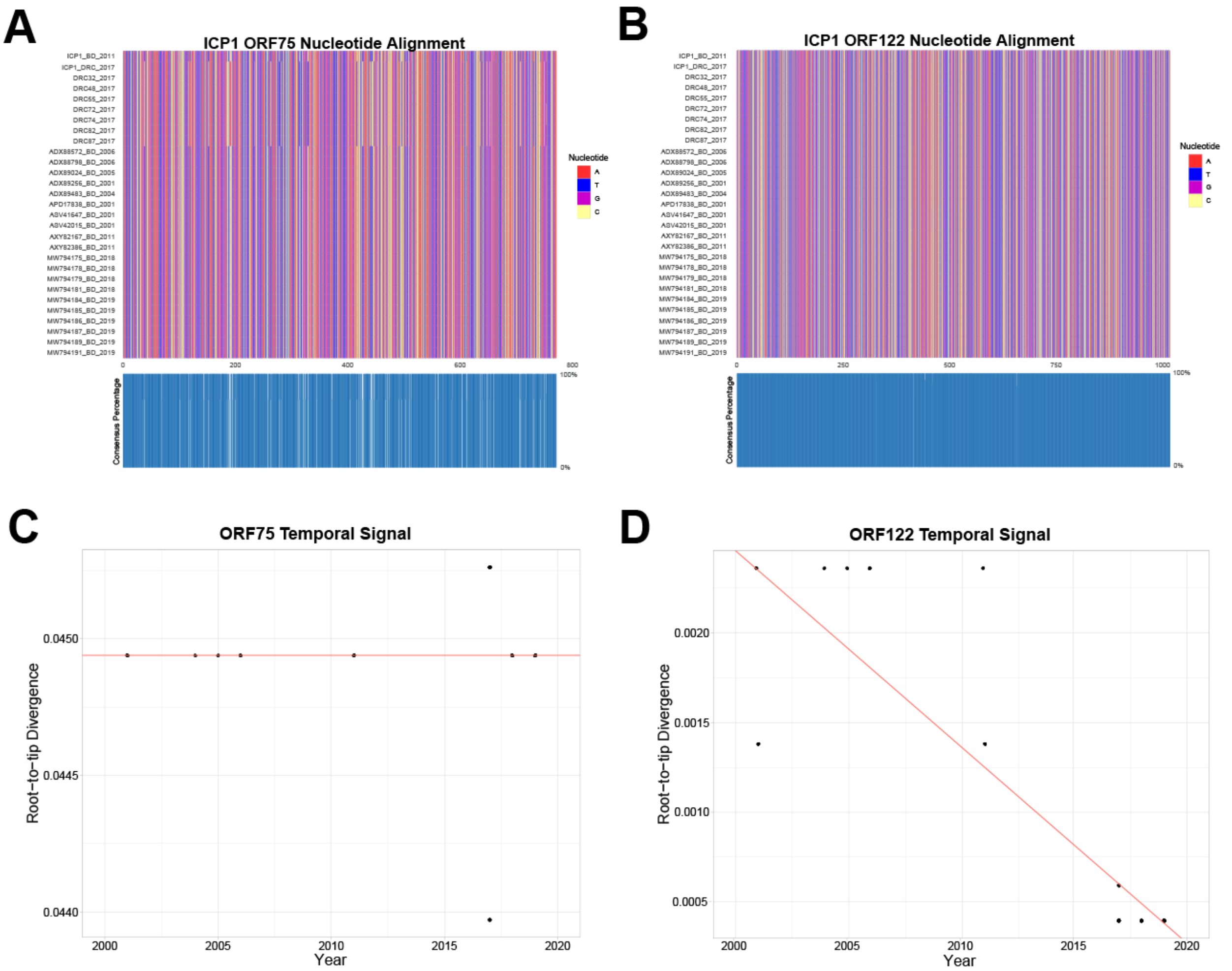

Fig S2. Multi-sequence alignment of ICP1 phage baseplate ORF75 **(A)** and capsular head ORF122 **(B)** nucleotide sequences. Sequences from both Bangladesh (BD) and Democratic Republic of Congo (DRC). Blue boxes at the bottom of A and B represent the percentage of the isolates that have the same nucleotide for that particular site. Temporal and divergence analysis of baseplate ORF75 (**C**) and capsular head ORF122 (**D**) nucleotide sequences from ICP1 strains isolated in Bangladesh.

Table S1. Microbiologic and molecular reagents

Reagent			
**Bacteria**	**Strain**	**Description**	**Reference**
V. cholerae	HC1037	O1 Ogawa serogroup, isolated from Haiti, SmR	(1)
**Bacteriophage**	**Strain (host)**	**Description**	**Reference**
ICP1	ICP1_2011_A (*V. cholerae* O1)	*Myoviridae*, isolated from Bangladesh	(2)
ICP1	ICP1_DRC_106 (*V. cholerae* O1)	*Myoviridae*, isolated from Goma, DRC	(3)
ICP2	ICP2_2004_A (*V. cholerae* O1 and non-O1)	*Podoviridae, isolated from Bangladesh*	(2)
ICP3	ICP3_2007_A (*V. cholerae* O1 and non-O1)	*Podoviridae*, isolated from Bangladesh	(2)

Table S2. List of immunogenic ICP1 core ORFs.

Predicted Protein	Function	Predicted Antigenicity (rank)[a]	Length (nt)	No of predicted epitopes[b]	Length (aa)
ORF66	hypothetical protein	1.03 (1)	228	3	75
ORF154	hypothetical protein	0.92 (2)	132	1	43
ORF158	hypothetical protein	0.83 (3)	258	3	85
ORF9	hypothetical protein	0.81 (4)	159	2	52
ORF206	hypothetical protein	0.77 (5)	219	3	72
ORF214	hypothetical protein	0.75 (6)	189	3	62
ORF75	putative baseplate assembly protein	0.73 (7)	774	7	257
ORF1	hypothetical protein	0.73 (8)	108	1	35
ORF195	hypothetical protein	0.71 (9)	162	2	53
ORF52	hypothetical protein	0.67 (10)	183	4	60
ORF122[c]	putative major head protein	0.54 (17)	1020	14	339

[a] Predicted antigenicity score of initially selected 11 ORFs by Vaxijen v2. The threshold for this model is 0.4 for a probable antigen. The rank is based on the antigenic score among 50 core ORFs (49 conserved core and 1 divergent core (c)) of ICP1 bacteriophage (17).
[b] Number of predicted B-cell epitopes by IEDB analysis. This analysis was done using Bepipred Linear Epitope Prediction 2.0 model.

Table S3. Molecular screening for predicted immunogenic targets in cholera and non-cholera stools.

**Bangladesh samples**	ID	ORF75[a]	ORF122[b]
V. cholerae negative samples (n=10)			
S1	RN22	-	-
S2	RN23	-	-
S3	RN24	-	-
S4	RN25	-	-
S5	RN26	-	-
S6	RN27	-	-
S7	RN28	-	-
S8	RN29	-	-
S9	RN30	-	-
S10	RN31	-	-
V. cholerae positive samples (n=2)			
S1 (ICP1-)	RN3	-	-
S2 (ICP1+)	VCP 12	**+**	**+**
**South Sudan samples**			
Random *V. cholerae* negative sample (n=2)			
S1	1001	-	-
S2	1002	-	-
V. cholerae positive sample (n=2)			
S1 (ICP1-)	1008	-	-
S2 (ICP1+)	1086	**+**	**+**

[a] represents the PCR amplification results for the target ORF75, a putative baseplate protein
[b] represents the PCR amplification results for the target ORF122, a putative ICP1 bacteriophage head protein.

REFERENCES

1. **Reyes-Robles T, Dillard RS, Cairns LS, Silva-Valenzuela CA, Housman M, Ali A, Wright ER, Camilli A.** 2018. *Vibrio cholerae* Outer Membrane Vesicles Inhibit Bacteriophage Infection. J Bacteriol **200**.
2. **Seed KD, Bodi KL, Kropinski AM, Ackermann HW, Calderwood SB, Qadri F, Camilli A.** 2011. Evidence of a dominant lineage of *Vibrio cholerae*-specific lytic bacteriophages shed by cholera patients over a 10-year period in Dhaka, Bangladesh. mBio **2:**e00334-00310.
3. **Alam MT, Mavian C, Salemi M, Tagliamonte MS, Paisie T, Cash MN, Angermeyer A, Seed KD, Camilli A, Maisha FM, Senga RKK, Morris JG, Ali A.** 2021. *Vibrio cholerae* multifaceted adaptive strategies in response to bacteriophage predation in an endemic region of the Democratic Republic of the Congo. medRxiv doi:10.1101/2021.07.30.21261389**:**2021.2007.2030.21261389.
